# Evaluation of Blood Coagulation by Optical Vortex Tracking

**DOI:** 10.3390/s22134793

**Published:** 2022-06-24

**Authors:** Jiaxing Gong, Yaowen Zhang, Hui Zhang, Qi Li, Guangbin Ren, Wenjian Lu, Jing Wang

**Affiliations:** 1 College of Life Science and Technology, Huazhong University of Science and Technology, Wuhan 430074, China; gjiaxing@hust.edu.cn (J.G.); yaowen.zhang@united-imaging.com (Y.Z.); zh2018@hust.edu.cn (H.Z.); liqi7012@hust.edu.cn (Q.L.); guangbin@hust.edu.cn (G.R.); m201971805@hust.edu.cn (W.L.); 2 Shenzhen Huazhong University of Science and Technology Research Institute, Shenzhen 518000, China

**Keywords:** blood coagulation, laser speckle, optical vortex, mean square displacement

## Abstract

Blood coagulation is a complicated dynamic process that maintains the blood’s fluid state and prevents uncontrollable bleeding. The real-time monitoring of coagulation dynamics is critical for blood transfusion guidance, emergency management of trauma-induced coagulopathy, perioperative bleeding, and targeted hemostatic therapy. Here, we utilize optical vortex dynamics to detect the blood coagulation dynamic process in a rapid and non-contact manner. To characterize the temporal changes in viscoelastic properties of blood during coagulation, we track the stochastic motion of optical vortices in the time-varying speckles reflected from 100 blood samples with varied coagulation profiles. The mean square displacement (MSD) of the vortices increases nonlinearly with time lag during blood coagulation reminiscent of the particles in viscoelastic fluids. The MSD curves with coagulation time are similar to the tracings of thromboelastography (TEG) during the blood coagulation. The retrieved coagulation parameters, such as reaction time and activated clotting time measured using the optical vortex method, exhibit a close correlation to those parameters acquired from TEG. These results demonstrate the feasibility of the optical vortex method for monitoring blood coagulation at the point of care. Our method is also applicable to measuring the viscoelasticity of complex fluids and turbid soft matters.

## 1. Introduction

The coagulation system is the body’s defense mechanism for maintaining normal blood flow and preventing blood loss [1,2,3]. Its abnormality can result from a multitude of conditions such as severe trauma, hypothermia, or surgery, and it can induce coagulopathy, including thrombocytopenia, subcutaneous hemorrhage, atherosclerosis, and even life-threatening bleeding [4,5,6,7]. The normal coagulation process depends on a delicate dynamic balance between coagulation, anticoagulation, and fibrinolysis [2,3,4]. Therefore, it is essential to monitor the coagulation status continuously for diagnosis, prophylaxis, and treatment of coagulation disorders.

However, coagulation is a cascade of complex biochemical processes involving interactions between platelets, red blood cells, and over a dozen coagulation factors, as well as two distinct coagulation pathways—endogenous and exogenous [8,9,10]. Conventional coagulation tests (CCTs) generally assess the coagulation status of patients by measuring the activated thromboplastin time (aPTT), prothrombin time (PT), platelet count (PLT), and fibrinogen levels, which require much time to separate plasma, platelets, and other components from whole blood [5,6,7]. Thus, traditional tests are not efficient enough to obtain the coagulation results, and the results are limited to coagulation parameters at a specific moment in time, which do not reflect the whole dynamics process of coagulation. In addition, numerical methods computational fluid dynamics (CFD) are developed to provide hemodynamic parameters and therefore help in the medical diagnosis of the coagulation process [11,12].

Currently, the primary clinical devices that can monitor the coagulation dynamics process are thrombelastography (TEG) and rotational thrombelastography (ROTEM). They evaluate the viscoelastic properties of a blood sample by applying a continuous oscillation to it and bringing it into contact with a metal wire to assess the formation, stabilization, and ultimately dissolution process [13,14,15,16]. While they overcome the drawbacks of CCTs, such as being unable to continuously monitor the whole process of coagulation, TEG and ROTEM, based on blood contact and mechanical excitation, delay clot formation and affect clot structure during measurements, leading to inaccurate coagulation results [15]. TEG and ROTEM are also less operable, more difficult to maintain, and more expensive due to their sophisticated components and complex construction.

Optical microrheological methods are frequently employed to investigate the rheological properties of soft tissues in a non-contactable manner. According to the generalized Stokes–Einstein relation (GSER), the viscoelasticity of a complex fluid can be obtained by calculating the MSD of the probe particles in it. Currently, optical techniques still need to track the motion of probe particles in surrounding environments, either directly or indirectly. In diffusing-wave spectroscopy (DWS) and dynamic light scattering (DLS), the viscoelastic properties are characterized by analyzing the temporal fluctuations of dynamic speckle patterns, $g_{2}\left(t\right)$, which is related to the random motion of scattering particles. When coherent light is irradiated on a multiple scattering medium, such as biological tissues, coherent light scattered by the scattering particles will form a spatially distributed pattern with bright and dark granular spots known as laser speckle. Laser speckle rheology (LSR) and coherence-gated DLS have been introduced to evaluate the viscoelasticity of biological tissues such as blood, articular cartilage, and mammary glands [17,18,19,20,21,22,23]. However, the measurement of intensity autocorrelation $g_{2}\left(t\right)$ in practice is highly complicated and entails taking into account static scattering, ordered or disordered motion of the scattering particles, the number of times the coherent light is scattered, etc. [24].

The laser speckle has a generic structure known as the optical vortex, which exists at an intensity of zero [25]. The real and imaginary parts at the location of the optical vortex are both zero, and the phase is ill-defined; thus, the optical vortex is also called a “phase singularity”. The phase around the optical vortex increases or decreases around the central spiral. Around a ‘positive’ vortex, the phase increases counterclockwise while the clockwise increasing phase indicates a ‘negative’ one. Moreover, the positive and negative optical vortices always create and annihilate in pairs. The optical vortex is an important research topic in the field of optics and gradually forms a branch of exotic optics, which has significant applications in many optical fields such as optical measurement [26,27,28,29], optical communication [30,31], and optical imaging [32,33,34].

In this study, we track the random motion of optical vortices to evaluate the viscoelastic properties of blood samples and thus assess blood coagulation dynamics. We investigate the relationship between the temporal change in the MSD of optical vortices and blood coagulation dynamics and retrieve the coagulation parameters, including reaction time (R-time), activated clotting time (ACT), and maximum amplitude of viscoelastic modulus of the thrombus (MA). To demonstrate the capability of the optical vortex method (OVM) for coagulation dynamics monitoring, we compare OVM and TEG results for more than 100 blood samples with different coagulation statuses. Finally, two-dimensional images of the blood viscoelasticity are obtained by scanning the surface of the blood sample at different stages of coagulation.

## 2. Materials and Methods

### 2.1. Porcine Blood Specimens and Coagulation Assay Preparation

We prepared over 100 whole blood samples with different coagulation statuses. The citrated whole blood was collected in a plastic bottle with 3.8% buffered sodium citrate with a citrate-to-blood ratio of 1:9. The heparinized whole blood was collected with 1% buffered sodium heparin with a heparin-to-blood ratio of 12.5 U/mL. After incubating the citrated blood for 5 min under 37 °C, 900–1300 µL of blood was transferred into vials pre-loaded with a kaolin buffer solution (Kaolin, Haemonetics Corp., Braintree, MA, USA). After 3 gentle vial inversions, 30–70 µL of 0.2 M calcium chloride (UR29102, Umibio, Shanghai, China) was added to the kaolin vial for activating coagulation. After another 3 gentle vial inversions, ~120 µL of kaolin-activated whole blood was immediately pipetted into an imaging chamber; in the meantime, an additional 360 µL of blood from the same tube was loaded into the sample cup of TEG (DRNX-III, Dingrun Medical Equipment Corp., Chongqing, China). The coagulation measurements were taken simultaneously in the OVM and TEG. The porcine blood samples exhibited a broad distribution of coagulation parameters, due to the difference in the type of anticoagulants, the ratio of the amount of blood to the amount of calcium chloride, and the storage time of the blood.

### 2.2. Experimental Setup

**Figure 1:**
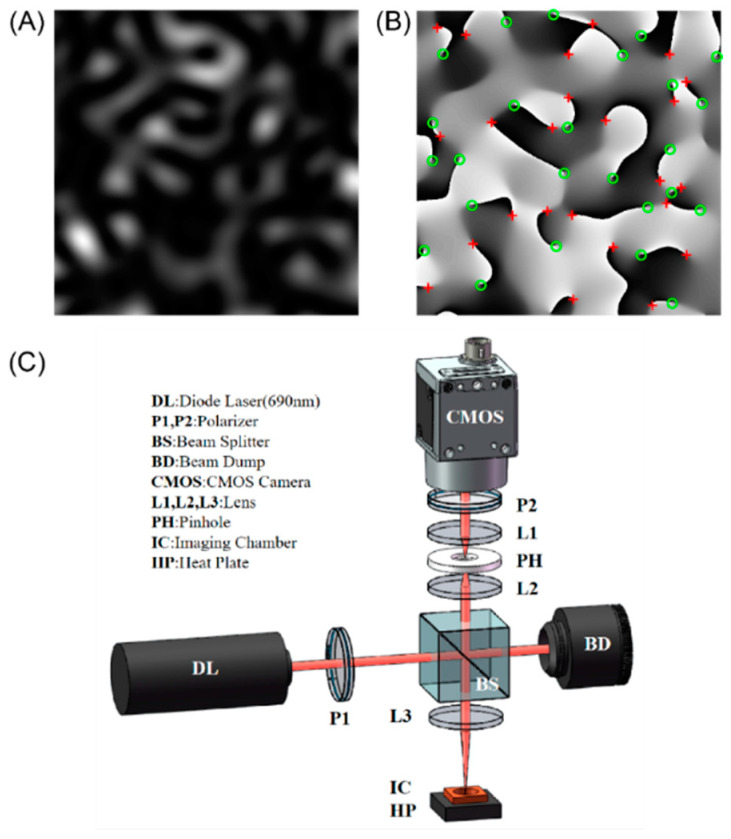
(**A**) The laser speckle pattern. (**B**) The pseudo-phase of the corresponding speckle pattern. The red crosses are positive optical vortices, and the green circles are negative optical vortices. (**C**) A schematic of the optical setup for acquiring dynamic speckle patterns.

The optical experimental setup shown in Figure 1C was used to capture time-varying laser speckle patterns of the prepared blood specimens. Light from a laser diode source (690 nm, 30 mW, Newport Corp., Irvine, CA, USA) is reflected off a beam splitter and focused on the top surface of the blood sample in an imaging chamber with a 5× objective lens. The heat plate maintained the temperature at 37 °C during the process of blood coagulation. Laser speckle patterns (Figure 1A), formed by the interference of the backscattered light passing through the medium, were acquired by a high-speed CMOS camera (acA2000-340kmNIR, Basler AG, Ahrensburg, Germany), together with a 4-f system and a linear polarizer. The pinhole was used to adjust the speckle size—approximately 4 × 4 pixels—to satisfy the Shannon sampling theorem. The second linear polarizer (P2) was used to eliminate the direct reflected light from the sample [29]. For each sample, measurements were taken for a total imaging duration of 20 min to evaluate the coagulation dynamics process. Every 20 s, one speckle frame sequence was captured at a frame rate of 800 frames per second (RoI: 192 × 192 pixels) for 0.5 s. Thus, a total of 60 time series of 400 speckle frames were collected during the blood coagulation.

To demonstrate the capability of the OVM for monitoring micro thrombosis, we performed the spatiotemporal analysis of the blood viscoelasticity during blood coagulation. The blood samples were scanned over an area of 1.7 × 1.7 mm^2^ with a motorized compact linear stage (M-VP-25XA-XYZR, Newport Corp., Irvine, CA, USA).

### 2.3. Calculating the MSD of the Optical Vortices during Blood Coagulation

Dynamic laser speckles are formed when the incident light is scattered by the moving scatterers in the whole blood, such as red blood cells, leukocytes, platelets, and fibrin monomers. For locating the optical vortex and reducing the complexity of the optical system, the captured speckle sequences are first converted into the complex analytic signal by Laguerre–Gaussian transform to obtain pseudo-phase $\hat{\phi }$ [35]:

(1) $$\tilde{I}\left(x,y\right)=\left|\tilde{I}\left(x,y\right)\right|\exp\left[i\hat{\phi }\left(x,y\right)\right]=I\left(x,y\right)*LG\left(x,y\right)$$

where I(x,y) is the original speckle intensity distribution and $\tilde{I}\left(x,y\right)$ represents the corresponding complex analytic signal; ∗ is the convolution operator; $\hat{\phi }\left(x,y\right)$ is the pseudo-phase representation; LG(x,y) represents the Laguerre–Gaussian function in the spatial signal domain, which can be calculated by the inverse Fourier transform of Equation (1):

(2) $$LG\left(f_{x},f_{y}\right)=\left(f_{x}+if_{y}\right)\exp\left(-\frac{f_{x}^{2}+f_{y}^{2}}{\omega^{2}}\right)=\rho \exp\left(-\frac{\rho^{2}}{\omega^{2}}\right)\exp\left(j\beta \right)$$

$\rho =\sqrt{f_{x}^{2}+f_{y}^{2}}$ and $\beta =\arctan\;\left(f_{y}/f_{x}\right)$ are the polar coordinates in the frequency domain. ω represents the bandwidth of the Laguerre–Gaussian function. The pseudo-phase $\hat{\phi }\left(x,y\right)$ can be calculated as follows:

(3) $$\hat{\phi }\left(x,y\right)=tan^{-1}\frac{Im\left\{\tilde{I}\left(x,y\right)\right\}}{Re\left\{\tilde{I}\left(x,y\right)\right\}}$$

Next, the optical vortices are located according to Equation (4):

(4) $$n_{t}\equiv \frac{1}{2\pi }\underset{c}{\oint }\nabla \hat{\phi }\left(x,y\right)d\vec{l}\equiv \frac{1}{2\pi }\iint_{D}\left(\frac{\partial^{2}\hat{\phi }}{\partial x\partial y}-\frac{\partial^{2}\hat{\phi }}{\partial y\partial x}\right)\partial x\partial y$$

where $n_{t}$ is the topological charge of the optical vortex,$\;\nabla \hat{\phi }\left(x,y\right)$ defines the local phase gradient, and the contour integral is taken over path l on a closed-loop *c* around the vortex. It is clear that $n_{t}\;$= 0 everywhere $\hat{\phi }$ is differentiable, except at the optical vortex locations where the phase is undefined. We used a two-dimensional phase unwrapping function to determine the position and topological charge of all the optical vortices with a window of 2 × 2 pixels at every point of the transformed pseudo-phase map. Only the topological charges of ±1 are recorded. 

**Figure 2:**
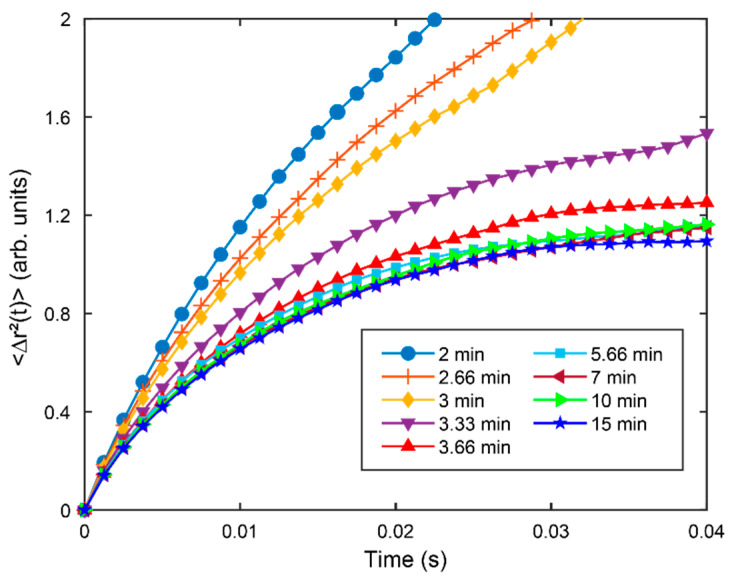
MSD curves of the optical vortex calculated at 2, 2.66, 3, 3.3, 3.66, 5.66, 7, 10, and 15 min during blood coagulation. It is obvious that the MSD curves decrease sequentially as the coagulation time proceeds, and the rate of decay slows down at the same time. A complete plateau level is reached after coagulation.

Mean square displacement is a common measure to investigate the dynamic behavior of particles. Here, we utilize the MSD of the optical vortex to estimate the evolution of blood coagulation dynamics:

(5) $$\langle \Delta r^{2}\left(t\right)\rangle ={\langle |\vec{r}\left(t_{0}+t\right)-\vec{r}\left(t_{0}\right)|}^{2}\rangle$$

where $\vec{r}\left(t_{0}+t\right)$ and $\vec{r}\left(t_{0}\right)$ represent the positions of the optical vortex at times $t_{0}+t$ and $t_{0}$, respectively, and 〈 〉 indicates ensemble averaging over the recording duration of a single speckle sequence. To calculate the MSD of the optical vortex according to Equation (5), we identified the corresponding optical vortices between two adjacent phase maps by restricting the search to not exceed the average diameter of speckle grain. MSD curves were calculated for each speckle sequence and plotted over the entire blood clot duration time, 0 < *t* ≤ 20 min (Figure 2).

### 2.4. Calculating Coagulation Parameters

**Figure 3:**
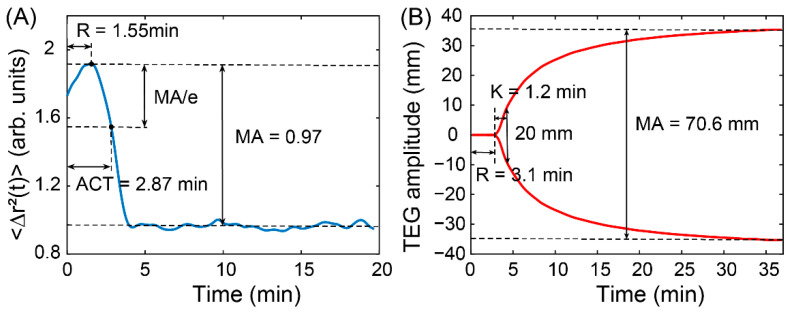
Coagulation time curves of the same sample measured by the OVM and TEG. (**A**) Coagulation trace of MSD over clotting time at a lag time of 0.02 s. (**B**) Corresponding coagulation profile measured by TEG. Differences in coagulation parameters result from different methods.

As previously described, the MSD of the optical vortex is able to characterize the viscoelasticity of the medium. We extracted the MSD values at the moment of 0.02 s, f=50 Hz, from all the speckle sequences to indicate clot viscoelasticity for the whole coagulation process. To calculate the coagulation parameters, the time course of the MSD data was displayed as a function of coagulation time (Figure 3A). Next, the time trace of blood coagulation dynamics measured using TEG during coagulation was compared with that measured using the OVM (Figure 3B). From the clotting curve by the OVM, the following coagulation parameters were extracted for comparison with TEG: reaction time (R-time), kinetic time (K), activated clotting time (ACT = R-time + K), and maximum amplitude (MA). In the OVM, the R-time was defined as the first distinct turning point on the clotting curve. The MA represents the maximum stiffness of the clot, as defined by the peak amplitude of the clotting curve. The ACT denoting the time to maximum fibrin formation was measured by calculating 1/*e* of the amplitude. We disregarded the angle calculation, which has the same meaning as indicated by the K value, i.e., both react to the rate of clot polymerization.

In the TEG method [14], the time between the start of data collection (at the SP time) to amplitude greater than 2 mm is defined as the reaction time (the R-time) that is deemed necessary to initiate the fibrin network formation. The K value is denoted by the time taken from the beginning of clot formation until the amplitude of the TEG output trace reaches 20 mm. The measurement of MA is similar to the OVM, but the MA of TEG is directly characteristic of the viscoelasticity of the blood sample.

## 3. Results

Figure 2 shows the MSD curves of the optical vortex reported at 2, 3, 3.33, 3.66, 5.66, 7, and 14 min following kaolin activation of blood coagulation. It is clear that the changing trend of MSD measured by the OVM is related to the viscoelastic properties of the clotting blood sample. As the coagulation reaction proceeds, the MSD of the optical vortex decreased sequentially. Following re-calcification and kaolin activation of coagulation factors, i.e., the initial stage of coagulation reaction, the MSD curves dropped at a fast rate (2–3.33 min). At 3.66 min after the onset of coagulation reaction, the MSD tends to level. A complete plateau level was reached at 5.66 min, and the subsequent measured MSD curves were maintained in its vicinity. Thus, after the blood coagulation process attained equilibrium characterized by a fully formed fibrin-platelet, the MSD of the optical vortex remained almost unchanged.

The blue curve in Figure 3A shows the results of the clotting curve measured by the MSD of the optical vortex in the porcine blood anticoagulated with sodium citrate during coagulation. Figure 3B shows the corresponding output measured for the same blood sample using TEG. As observed, the trend in the clot viscoelasticity profiles measured by the OVM closely resembles the TEG trace. Before the reaction time of coagulation, MSD exhibited only a slight change (from 1.7 at 0 min to 1.9 at 1.55 min), while the amplitude of TEG remained at 0 (from 0 min to 3.1 min). This indicates that blood initially exhibits the characteristics of a viscous material with a low viscoelastic modulus. During the progression of coagulation, the MSD decayed rapidly from 1.9 at 1.55 min to 0.96 at 4.15 min. However, the amplitude of TEG grew slower from 0 mm at 3.1 min to 35.3 mm at 36 min. This indicates that the optical vortices travel slower and slower while the viscoelasticity of the blood increases gradually. Following stabilization of the coagulation process at later clotting times, both MSD and TEG amplitude approached saturation levels. It is worth noting that the measured total clotting time and R-time by the OVM are less than those by TEG. This is likely due to the higher sensitivity in detecting the optical vortex movements resulting from alterations in clot modulus during coagulation. Furthermore, the principal drawback of TEG is that the strain amplitude associated with its operation is uncontrolled and gradually decreases during measurements, which can substantially delay clot formation and modify clot structure throughout coagulation [16,36].

**Figure 4:**
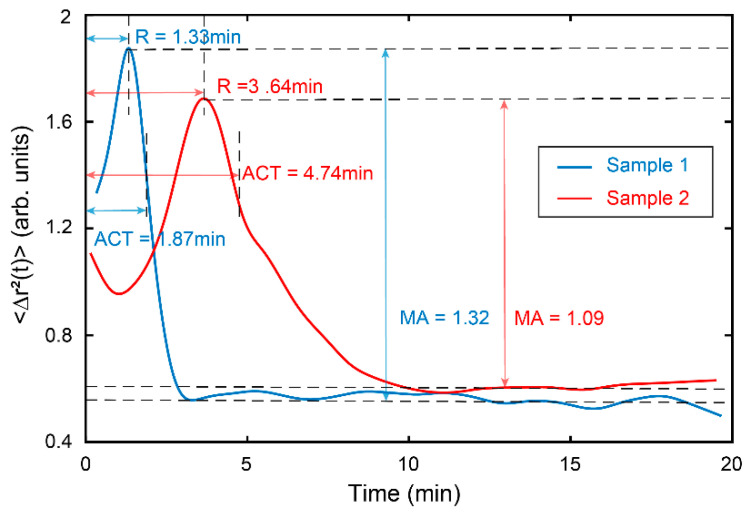
Time traces of coagulation measured from the blood samples by using 35 μL (red line) and 70 μL (cyan line) 0.2 M calcium chloride to initiate the blood coagulation. It is clear that both R-time and ACT were significantly prolonged when calcium chloride used was decreased from 70 μL to 35 μL. In contrast, the MA decreased as the calcium chloride used decreased.

Figure 4 shows coagulation experiments using 35 µL and 70 µL 0.2 M calcium chloride, with other conditions such as the amount of blood used and types of anticoagulants remaining consistent. As an essential factor in the coagulation reaction, the amount of calcium directly affects the whole coagulation process. Thus, different coagulation parameters were obtained by controlling the amount of calcium used. As shown in Figure 4, R-time is 1.33 min and ACT is 1.87 min when using 70 µL calcium chloride. When the calcium chloride used was decreased to 35 µL, both R-time and ACT were significantly prolonged to 3.64 min and 4.74 min, respectively. This indicates that increasing the amount of calcium chloride within a certain range can accelerate the clotting reaction. Meanwhile, the amplitude (MA = 1.32) measured by 70 µL calcium chloride is higher than it (MA = 1.09) by 35 µL calcium chloride. To explain this, it could be suggested that more calcium chloride led to more thrombin, which can convert fibrinogen to fibrin, enhancing the formation of a fibrin clot.

**Figure 5:**
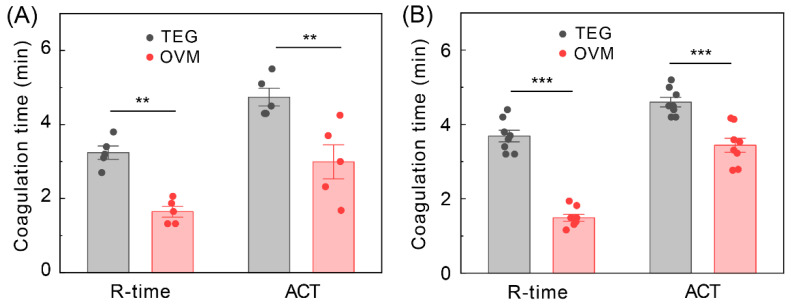
Coagulation profiles of blood with the same initial coagulation conditions. (**A**) Blood anticoagulated with sodium citrate. The analyzed blood samples with different storage times, 2~14 days, n = 5. (**B**) Blood anticoagulated with sodium heparin. The analyzed blood samples with different storage times, 2~22 days, n = 8. Mean ± SD. ** *p* < 0.01, *** *p* < 0.001; two-way ANOVA followed by Tukey post hoc test.

We analyzed the coagulation profiles of several blood samples with the same initial conditions to validate the robustness of our method. Figure 5 shows the results of 5 independent experiments using the citrated blood sample and 8 independent experiments using the heparinized blood sample, both reactivated by 40 µL calcium chloride. The comparable standard deviations of the coagulation parameters R-time and ACT for the blood samples between TEG and OVM show good repeatability. The variations may result from the sample-to-sample fluctuations, diverse storage time of the blood, and laboratory test conditions. Moreover, we compared the R-time and ACT measured by our OVM with those measured by TEG, suggesting significant reductions in coagulation time, *p* < 0.01 for citrated blood samples and *p* < 0.001 for heparinized blood samples. OVM measures the coagulation profiles in a non-contact way, while TEG’s compelling direct contact to the blood may destroy the cross-linking structures during blood coagulation, thus resulting in the measurement time reduction [36]. The less measurement time in OVM than in TEG dictates the potential of our method for rapid real-time bedside tests. The results of our method and TEG do not show a significant difference for different anticoagulants, sodium citrate, and sodium heparin, and it requires additional work to explore the effects of anticoagulants in blood dynamics monitoring.

**Figure 6:**
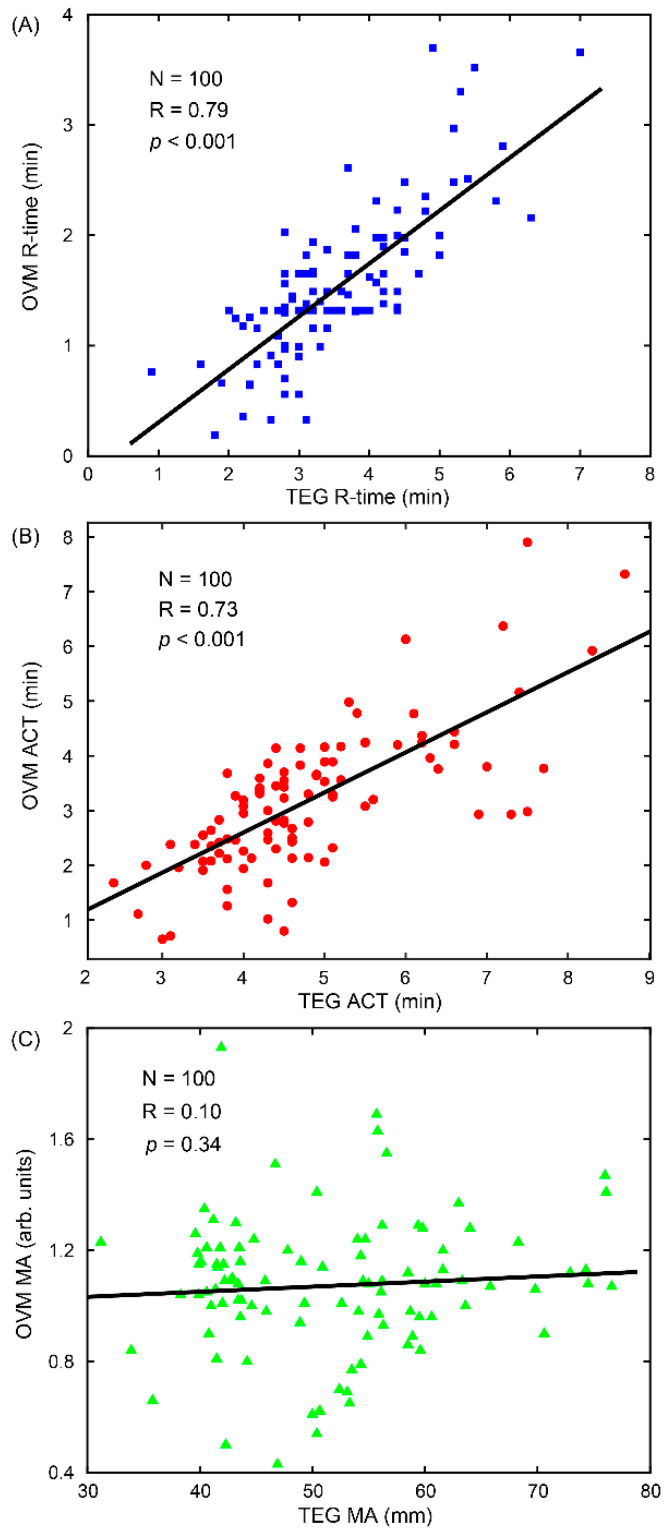
A comparison of variable coagulation parameters acquired from our technique versus TEG. (**A**) R-time, (**B**) ACT-time, and (**C**) MA obtained with the OVM and TEG with 100 different porcine blood samples. R-time and ACT-time showed a strong positive correlation with the gold standard but MA presented a weak correlation, which is worthy of discussion.

In Figure 6, the coagulation parameters, R-time, ACT, and MA, measured from the OVM of 100 different porcine blood experiments, are compared with the corresponding parameters measured by the TEG device using parametric Pearson’s correlation coefficient, R, analysis. The coagulation parameters were measured by the OVM with a relatively wide distribution of values such as R-time ranging from 0.19 to 3.7 min, ACT ranging from 0.65 to 7.9 min, and MA ranging from 0.43 to 1.93. Meanwhile, the corresponding coagulation parameters were measured by TEG, R-time ranging from 0.9 to 7 min, ACT ranging from 2.4 to 8.7 min, and MA ranging from 31.2 to 76.6 mm. For the first two coagulation parameters, R-time and ACT, a strong positive correlation between the OVM and TEG was observed (*p* < 0.001), confirming the accuracy of measurement to clotting time. The OVM parameters R-time and ACT bore a linear relationship with the corresponding TEG values with correlation coefficients, R, of 0.79 and 0.73, respectively. In contrast, for the MA parameter, a significant correlation between the OVM and TEG was not found in the experiment. Many possible factors led to the deviation, one of the most likely being due to differences in the moduli measured for clots formed under quiescent conditions by the OVM, versus agitation conditions of mechanical strain by TEG, as discussed below [37].

**Figure 7:**
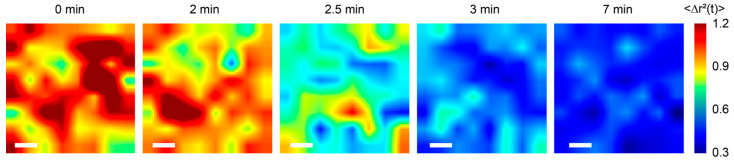
Spatial maps of MSD of the optical vortex at 0, 2, 2.5, 3, and 7 min after kaolin activation. Significant MSD values change at 2–2.5 min and maintain a low level over 7 min in the porcine blood. Scale bar: 200 μm.

We also analyzed the spatiotemporal behavior of optical vortices to present the spatial distribution of viscoelastic properties of blood during coagulation. The two-dimensional color maps of MSD in Figure 7 were obtained at minutes 0, 2, 2.5, 3, and 7 after kaolin activating blood coagulation. The MSD of the vortex is inversely related to the viscoelastic properties, similar to the generalized Stokes–Einstein relation. The downward tendency of MSD reflected the time-dependent alteration in the viscoelastic properties of blood during coagulation. The micro clots were appealing in the initial stages of coagulation (light blue islands in Figure 7, 2–2.5 min), then developed to a stabilized state. Meanwhile, mechanical testing techniques such as TEG and ROTEM are incapable of detecting the creation of these early micro clots, as induced shearing of the sample is suspected of destabilizing the initial fibrin structure and impairing clot formation.

## 4. Discussion

In this study, we have proposed a novel method to monitor the dynamics process of blood coagulation by gauging the viscoelasticity of the blood from the stochastic motion of the optical vortex. The results show that the dynamic behavior of the optical vortex under restrictions during blood coagulation is similar to the scattering particles in a viscoelastic medium. Moreover, comparative experimental results of the OVM and TEG demonstrated the capability of the OVM to evaluate blood coagulation status. Blood coagulation parameters such as R-time and ACT measured by the OVM showed a strong correlation with TEG. The time required for global coagulation profiling by the OVM is significantly less than that required by TEG when blood is activated with the same coagulation activator, as shown in Figure 3. In addition, the elementary optical devices and low interference with the sample are also essential advantages for efficiently assessing a patient’s blood coagulation status.

Laser speckle is a granular intensity pattern produced by the interference of coherent light undergoing multiple scattering from the randomly distributed particles [38]. Laser speckle techniques have been applied to investigate biomedical processes for many years. For example, laser speckle contrast imaging has been widely accepted as an effective method for imaging the blood flow and blood perfusion by measuring the speckle contrast spacetime variations induced by the fast motion of red blood cells in superficial blood vessels in the brain [24]. In biomechanics evaluation, the range of particle motion is one of the primary parameters, which is governed by the viscoelastic susceptibility of the microenvironment surrounding the particles. Laser speckle technology has been successfully used to measure the viscoelastic properties of various tissues, such as coronary plaques, human osteoarthritic knee cartilage, and breast tumors [39]. It utilizes the temporal intensity autocorrelations of dynamic speckle patterns resulting from the random motion of light scattering particles in confined geometries. Therefore, the random optical phase distribution of numerous scattered light waves arising from the motion of scattering particles can be accessed to recover the structured knowledge of the biological tissue.

It is thus possible in principle to characterize the interaction of the wave through the sample by tracking the displacement of specific features of the speckle pattern in time [26,40]. As the generic and distinctive structures of a speckle pattern, optical vortices or phase singularities can be used to characterize the viscoelastic properties of the tissue [29]. Furthermore, the density of the optical vortex is the same order as the speckle spots in a fully developed speckle, and the vortices can determine the structure of the speckle grains since they instruct the sizes and locations of zones of constant phase [41]. Researchers have proposed exploiting first-order statistics of optical vortex motion to measure nanometric displacement and analyze biological kinematic information [27,42]. Like laser speckle rheology and dynamic light scattering investigating the motion of light scatterers, we can explore the second-order stochastic information such as the mean square displacement of the optical vortex to obtain the dynamic features of the medium.

Here, we investigate the accuracy and sensitivity of the method to estimate the coagulation status of whole blood samples. In unclotted blood, RBCs and platelets are the most dominant scattering particles, with random and rapid motion, maintaining stable optical properties. When coagulation is initiated, soluble fibrinogen is transformed into insoluble fibrin and polymerizes with activated platelets and RBCs, which leads to scattering particles undergoing restricted Brownian excursions. The displacement of scattering particles defined by the MSD is related to the viscoelastic modulus of the surrounding environment via the GSER as follows [43,44]:

(6) $$|G^{*}\left(\omega \right)|=\frac{K_{b}T}{a\pi \langle \Delta r^{2}\left(1/\omega \right)\rangle \Gamma \left[1+\alpha \left(\omega \right)\right]}$$

where $K_{b}$ is the Boltzmann constant, T represents the Kelvin scale of temperature; a is the average radius of the light scattering particles; $\langle \Delta r^{2}\left(1/\omega \right)\rangle$ defines the MSD of the light scattering particles at time 1/ω; Γ denotes the gamma function. α(ω) is given by

(7) $${\left|\alpha \left(\omega \right)=\frac{dln\langle \Delta r^{2}\left(t\right)\rangle }{dlnt}\right|}_{t=1/\omega }$$

However, the average radius of the light scattering particles changes continuously with the blood coagulation. It is difficult to obtain the quantified values of the viscoelastic modulus using optical methods based on microrheology. Furthermore, the methods require measurement of the scattering coefficient, *μ_s_*, which evolves continuously as coagulation progresses in the blood sample. To avoid omitting many parameters, the MSD of actual scattering particles is represented by the MSD of the optical vortex. As a virtual particle, it makes little sense to measure its average radius. Its MSD can be calculated directly by the method mentioned above and used to characterize the viscoelasticity of the medium, which is verified in the previous study.

To calculate the MSD of the optical vortex accurately, the acquisition frame rate should be selected suitably. Due to the rapid change of laser speckle during blood coagulation, the low acquisition frame rate is unable to capture the trajectories of the optical vortex. In this study, the high frame rate of 800 frames per second was chosen for capturing the rapid speckle fluctuations induced by a cascade of coagulation processes. Although higher frame rates are theoretically more useful for capturing the trajectory of the optical vortex, the acquisition frame rate is limited by some hardware parameters such as spatial resolution and exposure time. Small spatial resolution may cause an insufficient number of the optical vortex to be ensemble averaged, resulting in unreliable MSD results. Exposure time directly affects the contrast of the image, which is important for the location of the optical vortex. While ensuring sufficient contrast in the image, the exposure time can be reduced by increasing the gain value, thus allowing for a higher frame rate, but this causes more noise and reduces the SNR. A significantly reduced SNR could cause trouble in locating and pairing the optical vortex. Thus, the balance between frame rate and other hardware parameters is essential for the accurate calculation of the optical vortex’s MSD.

The captured speckle intensity distribution is represented by real values, while the optical vortex is located in the corresponding phase map. It is common practice in physics and engineering to use complex-valued signals to represent the real-valued signals. A true phase map cannot be obtained by using the simple optical device in this study. We have demonstrated that the MSD of the optical vortex calculated by using pseudo-phase has a strong correlation with it by using real phase in the previous simulation experiments. Therefore, the Laguerre–Gaussian transform was subsequently applied to the 2-D speckle pattern, acquiring the pseudo-phase according to Equation (3) in this study. Several reports have shown that the Riesz transform and Hilbert transform could also be used for obtaining the pseudo-phase, but the Laguerre–Gaussian transform with some irreplaceable advantages was chosen eventually for this study. It can readily be seen from Equation (2) that the amplitude of the Laguerre–Gaussian function is doughnut-like, which can suppress high-frequency components of unstable phase singularities, thus serving as a band-pass filter. As the bandwidth of this filter, ω can be used to adjust the density of phase singularities in the pseudo-phase by controlling the average speckle size. The high density of the optical vortex may cause the recurrence and collisional disappearance of the optical vortex under the same position, resulting in more pairing errors. In the other case, the insufficient number of the optical vortex can lead to inaccurate statistical results. Thus, it is necessary to choose the suitable value of ω for the calculation of MSD.

The annihilation of optical vortices resulting from the collision of positive and negative vortices is a disruption for the statistical calculation of MSD. The random distribution property of the optical vortex originating from the random field of laser speckle could be the main cause of this problem [45,46]. During blood coagulation, the rapid change in laser speckle raises the chance of optical vortex collisions, causing the optical vortex to only survive for a short time. Thus, when the statistical time for the optical vortex’s motion increases, the MSD will be unreliable because of the insufficient quantity of the optical vortex. As shown in Figure 2, we conducted a statistical analysis of all sequences with an acquisition time of 0.5 s. The MSD trace that can characterize the viscoelasticity of the blood was obtained at the statistical time, *t* = 0.04 s. Moreover, in the initial stage of coagulation reaction (2–3 min), the existence time of the optical vortex trajectories becomes shorter due to faster fluctuations of laser speckle. Here, the MSD values at the moment of 0.02 s were chosen eventually to indicate clot viscoelasticity for the evaluation of the whole coagulation process. The coagulation parameters, R-time and ACT, which were defined by the OVM presented a strong correlation with corresponding TEG values, while the MA parameter showed a weak correlation. It may be explained by the differences in the measurement mechanisms of the two methods. The OVM is an optical method that can measure the blood viscoelasticity without mechanical contact during coagulation, causing less mechanical disruption of the clot. In contrast, in TEG, oscillatory shear stress is applied for measuring the viscoelasticity throughout the whole coagulation process, disrupting the clot structure during polymerization and stabilization, thereby having an obvious impact on MA. Furthermore, the MA parameter is related to stiffness measured for clots formed under quiescent conditions, which is connected with the amount of calcium chloride. In the OVM, *t* = 0.02 s, corresponding to a high shearing frequency of ω = 50 Hz was used for the measurement of different reagent ratios, while the low shearing frequency of TEG works also only on the measurement of a fixed ratio reagent. Thus, the absence of a dose-dependent modulation is a possible cause of low MA correlation.

The studies described here are mainly limited by the variations of optical properties during blood coagulation. Optical vortex behavior in dynamic laser speckles is sensitive to the random motion of the scattering particles, and therefore related to the viscoelastic properties of the surrounding medium. However, speckle fluctuations are influenced not only by sample mechanics, but also by changes in optical properties of blood, the reduced scattering coefficient $\mu_{s}^{\prime }$, and absorption coefficient $\mu_{a}$. The absorption coefficient stays almost the same due to the constant concentration of hemoglobin. During the initial stage of coagulation, platelet aggregation reduced the amount and enlarged the size of scatterers, which led to a reduction in the scattering coefficient. Then the occurrence of fibrin polymer networks induced more scattering centers and resulted in the increment of $\mu_{s}^{\prime }$. The change of $\mu_{s}^{\prime }$ almost vanished when the fibrin stabilized. In clinical, the variations of optical properties of blood may have a tiny influence on our results, like in laser speckle rheology [21,23]. Another experimental discrepancy of our technique is the mechanical vibration from the complex environments in clinical diagnosis. Because OVM investigations use whole blood, like other viscoelastic tests, the results are likely to deviate from laboratory testing under circumstances such as hemodilution and platelet dysfunction.

In the past, devices such as TEG and ROTEM have been investigated for efficient detection of coagulation at the point of care. In these methods, a cylindrical cup containing a whole blood sample oscillates continuously and a pin on a torsion wire is suspended in the blood. The torsion wire is a sensor that can transmit the changes in blood viscoelasticity to an electromagnetic transducer, implementing a coagulation detection function [14]. However, continuous mechanical oscillation will disrupt the micro clot structure in the early stages of clotting, enabling them to only evaluate the viscoelastic modulus of larger clots effectively. Thus, the methods described above are less sensitive to tiny and local changes in blood viscoelasticity. By comparison, in the OVM, the measurements of the optical vortex are related to the nanometer-scale motion of scattering particles, related to the speckle fluctuations [47,48]. Therefore, the new approach is theoretically sensitive enough to the small changes in clot viscoelasticity and can measure microclots earlier than TEG and ROTEM. In the study, the R-time measured by the OVM ranged from 0.19 to 3.7 min, while the corresponding value measured by TEG ranged from 0.9 to 7 min. Likewise, the ACT measured by the OVM (0.65–7.9 min) is much less than TEG (2.4–8.7 min). The results demonstrated a shorter clotting time (R-time and ACT) compared with TEG, which proves the high sensitivity to incipient micro clots in the OVM.

## 5. Conclusions

In this study, we validated the capability of the OVM for the non-contact and rapid monitoring of blood coagulation dynamics at different stages of coagulation, using only a few drops of whole blood. The low cost and compact setup that can be easy to operate without special treatment is also a noteworthy advantage for assessing a patient’s blood coagulation status at the bedside. In the future, clinical experiments will be conducted for further correlation studies to validate the potential as a miniaturized medical device for coagulation testing. Our technology extends the optical micro-rheology to turbid fluids like blood by using optical vortices as virtual probes instead of real particles. We believe that our approach can find applications in the viscoelasticity measurement of various complex fluids and biological tissues.

## Data Availability

The data presented in this study are available on request from the corresponding author.
